# Bariatric Surgery Outcomes in Patients with Inflammatory Bowel Disease in the United States: An Analysis of the Nationwide Readmissions Database

**DOI:** 10.1007/s11695-024-07111-w

**Published:** 2024-02-27

**Authors:** Noah C. Wilson, Danielle B. Dilsaver, Ryan W. Walters, Kalyana C. Nandipati

**Affiliations:** 1 School of Medicine, Creighton University, 2500 California Plaza, Omaha, NE 68178 USA; 2 Department of Clinical Research and Public Health, School of Medicine, Creighton University, 7710 Mercy Road, Education Building, Suite 502, Omaha, NE 68124 USA; 3 Department of Surgery, School of Medicine, Creighton University, 7710 Mercy Road, Education Building, Suite 501, Omaha, NE 68124 USA

**Keywords:** Inflammatory bowel disease, Crohn’s disease, Ulcerative colitis, Bariatric surgery, Sleeve gastrectomy

## Abstract

**Purpose:**

Bariatric surgery has been reported to produce durable weight loss in the management of obesity; sleeve gastrectomy (SG) is the most common bariatric procedure. Obesity is a common comorbidity of inflammatory bowel disease (IBD), and the impact of IBD on short-term SG outcomes has not been widely reported. This study assessed whether IBD was associated with adverse post-SG outcomes.

**Materials and Methods:**

Hospitalizations of patients undergoing SG in the United States were identified using the 2010–2020 Nationwide Readmissions Database (NRD) and stratified by IBD diagnosis. The SG cohort was propensity-matched based on age, biological sex, body mass index (BMI), comorbid diabetes, hypertension, depression, chronic obstructive pulmonary disease, and discharge in quarter four. Primary aims were to compare in-hospital mortality, post-operative complications, and all-cause 90-day readmission between patients with and without IBD. Secondary outcomes were length of stay (LOS) and total hospital cost.

**Results:**

A total of 2030 hospitalizations were matched. The odds of complication were 48% higher for hospitalizations of patients with IBD (11.1% vs. 7.8%; aOR 1.48, aOR 95% CI 1.10–2.00, *p* = .009). The most common complication was nausea (4.9% vs. 3.7%, *p* = .187). No statistically significant difference was observed for all-cause 90-day readmissions, LOS, or hospital cost.

**Conclusion:**

Hospitalizations of patients with IBD who underwent SG experienced significantly higher post-operative complication rates. However, the similar lengths of stay and readmission rates compared to propensity-matched SG hospitalizations without IBD suggest many complications were minor. SG remains a safe weight loss procedure for patients suffering from IBD and obesity.

**Graphical Abstract:**

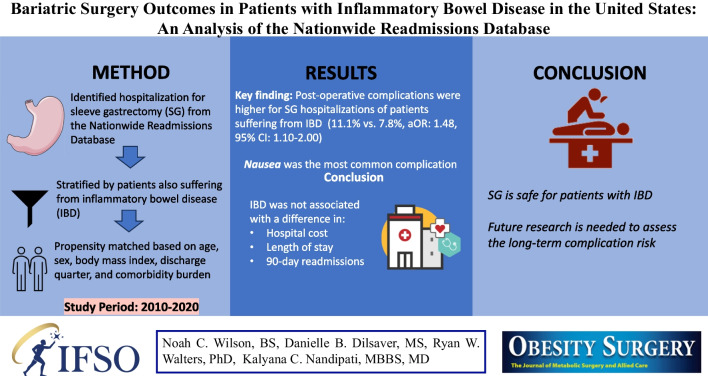

**Supplementary Information:**

The online version contains supplementary material available at 10.1007/s11695-024-07111-w.

## Introduction

In the United States (US), the prevalence of obesity is at an all-time high; more than 40% of American adults suffer from obesity based on a BMI ≥ 30.0 kg/m^2^, an increase from 30.5% in 1999 [1]. Obesity has been linked to adverse health outcomes such as hypertension, coronary artery disease, sleep disorders, and cancer [2]. There is also a significant economic burden associated with obesity, which accounted for $172.74 billion of annual healthcare-related expenditures among adults from 2011 to 2016 [3].

In parallel with obesity, cases of inflammatory bowel disease (IBD)—which includes Crohn’s disease (CD) and ulcerative colitis (UC)—have also shown increasing prevalence [4, 5]. Patients suffering from obesity also frequently suffer from IBD; approximately 1 in 3 patients with a diagnosis of IBD suffer from obesity [6]. This observation, combined with the parallel increases in cases of obesity and IBD, has led to speculation that there may be a link between the two epidemics. Specifically, it has been hypothesized that IBD and obesity may share a similar disease process mediated by a common set of proinflammatory molecules [7, 8]. Thus, treating a patient’s obesity may positively impact the course of IBD [9].

Bariatric surgery has been considered the main therapeutic option for managing obesity, especially in patients for which other nonsurgical interventions have proven ineffective. Bariatric procedures have been repeatedly shown to produce greater weight loss than nonsurgical management of obesity [10], with sleeve gastrectomy (SG) among the most popular bariatric surgeries worldwide [11]. In a small retrospective series concerning the management of patients with IBD, SG was reported to have fewer complications compared to Roux-en-Y gastric bypass (RNY) [12]. Yet, there is still a paucity of data on in-hospital post-operative outcomes and risk of rehospitalization for patients with IBD undergoing SG. The current study addresses this gap by evaluating the post-operative safety of SG in patients carrying an IBD diagnosis. The Nationwide Readmissions Database (NRD) was used to compare differences in in-hospital mortality, in-hospital post-operative complications, and all-cause 90-day readmission rates between patients undergoing SG with and without comorbid IBD in the US.

## Materials and Methods

### Data Source

Study data were abstracted from 2010 through 2020 Nationwide Readmissions Database (NRD). The NRD is an inpatient care database in the United States (US) and estimates approximately 35 million yearly hospital discharges [13]. The NRD is part of the Healthcare Cost and Utilization Project (HCUP) and is sponsored by the Agency for Healthcare Research and Quality (AHRQ). The NRD is a representative sample of the US population and includes inpatient hospital stays irrespective of expected payer. Notably, the NRD captures in-hospital mortality and repeat hospital visits within a calendar year. The NRD includes patients with and without repeat hospital visits. The NRD only captures in-hospital events and does not provide outpatient outcomes (e.g., outpatient mortality). The NRD is de-identified and HIPAA-compliant. The Institutional Review Board at Creighton University (InfoEd record number: 2003782) acknowledged this study as Not Human Subjects Research.

### Identification of Hospitalizations for Sleeve Gastrectomy and Irritable Bowel Disease

To identify sleeve gastrectomy (SG) hospitalizations, we first identified hospitalizations of patients undergoing obesity procedures using Medicare Severity Diagnosis Related Groups (MS-DRG: 619, 620, and 621) [14]. Next, these obesity surgery hospitalizations were further identified as SG procedures as indicated by ICD-9 and ICD-10 procedure codes (ICD-9: 43.82 and ICD-10: 0DB64Z3). After identifying these index SG hospitalizations, the hospitalizations were stratified by whether the patient also suffered from IBD as indicated by ICD-9 and ICD-10 diagnosis codes (CD: 555.0, 555.1, 555.2 555.9, K50.-; and UC: 556.0–556.6, 556.8, 556.9, K51.-). Lastly, SG hospitalizations were excluded if the patient was younger than 18 years of age or had previous bariatric surgery as indicated by ICD-9 and ICD-10 diagnosis codes (ICD-9/10: V45.86, V45.75, Z98.84, Z90.3).

Patient-specific covariates were extracted for index hospitalizations meeting inclusion criteria; patient-specific covariates included age, biological sex, and body mass index as indicated by ICD-9 and ICD-10 diagnosis codes (see Supplemental Table SI). Additionally, Elixhauser-refined comorbidities were extracted for index SG hospitalizations. Elixhauser comorbidities are a set of pre-existing variables within the NRD that are based on secondary diagnoses that co-exist at the time of hospitalization. Elixhauser comorbidities were selected because they relate to healthcare resource allocation (e.g., length of stay) and outcomes (e.g., in-hospital mortality). The NRD and other databases within the HCUP family demonstrate good validity to support the use of Elixhauser comorbidities to identify co-existing conditions at the time of hospitalization [15]. The following Elixhauser-refined comorbidities were extracted: acquired immunodeficiency syndrome (AIDS), alcohol abuse, rheumatoid arthritis/collagen vascular disease, metastatic cancer, depression, diabetes with and without chronic complications, drug abuse, hypertension with and without chronic complications, lymphoma, chronic pulmonary disease, peripheral vascular disorders, and hypothyroidism [15].

### Study Outcomes

Primary outcomes included in-hospital mortality, post-operative in-hospital complications, and all-cause and cause-specific 90-day readmissions. Secondary outcomes included index hospitalization length of stay (LOS) and hospital cost that was inflation-adjusted to mid-year 2020 US dollars [16]. Post-operative complications were provided as a composite and included bariatric-specific complications, general complications, ulceration, organ complications, hemorrhage, and infection. Complications were identified using ICD-9 and ICD-10 diagnosis codes (see Supplemental Table SI). Specific to 90-day readmission analysis, the NRD only follows patients for a calendar year and does not allow for readmission analysis across years. As such, to allow for complete 90-day post-discharge follow-up for all patients, patients discharged within 90 days of the end of the calendar year were excluded. More specifically, index hospitalizations were excluded if the patient died or was discharged in October, November, or December. Cause-specific readmission rates were identified by the primary discharge diagnosis of the repeat hospital visit using MS-DRG codes [14].

### Statistical Analysis

Descriptive statistics were stratified by whether the patient suffered from IBD. Categorical covariates were presented as percent and compared using Rao-Scott chi-square test which is a designed-adjusted chi-square test that incorporates the sampling design of the NRD [17]. Continuous covariates were presented as median and interquartile range. Logistic regression models were estimated to evaluate whether the odds of in-hospital post-operative complication and the odds of all-cause 90-day readmission differed by whether the patient suffered from IBD. Lognormal models were estimated to evaluate differences in the LOS and hospital cost by whether the patient suffered from IBD. To assess whether the complication rate differed between patients suffering from CD and patients suffering from UC, a logistic regression model was estimated.

To adjust for baseline differences and to account for selection bias between SG hospitalizations of patients suffering from IBD and SG hospitalizations of patients not suffering from IBD, index hospitalizations were propensity score–matched based on age, biological sex, body mass index ranges, and comorbid conditions that included diabetes, hypertension, depression, and chronic obstructive pulmonary disorder. For the 90-day readmission analysis, we ensured there was the same number of patients suffering from IBD and patients not suffering from IBD by matching based on whether the patient was discharged in the fourth quarter of a given calendar year (aka, October, November, or December). As discussed in the study outcomes, the NRD only follows patients for a calendar year and does not allow for readmission analysis across years. To allow for complete 90-day post-discharge follow-up for all patients, patients discharged in October, November, or December were excluded from the 90-day readmission analysis. Greedy nearest neighbor 1:1 matching was used; biological sex, body mass index, discharge quarter four, and comorbidities were exact-matched; age was matched to minimize Mahalanobis distance between hospitalizations of patients suffering from IBD and hospitalizations of patients not suffering from IBD. Mahalanobis distance is a multivariate distance metric that is useful for identifying outliers and reducing bias in propensity score matching; briefly, the Mahalanobis distance is the multivariate distance of an observation from the mean of all variables simultaneously (aka, centroid). Match quality was assessed through the distribution of propensity scores and standardized difference scores (± 0.10 indicated imbalance and poor match quality). All outcome analyses were based on the matched cohorts and accounted for the NRD sampling design with two-tailed *p* < 0.05 used to indicate statistical significance. All analyses were conducted using SAS v. 9.4.

## Results

In the United States, from 2010 to 2020, sleeve gastrectomy (SG) was the most common bariatric procedure for patients suffering from IBD; SG accounted for 96.9% of bariatric procedures. Overall, there were an estimated 914,345 hospitalizations of patients suffering from obesity who underwent SG (unweighted *N* = 492,438; Table SII), of which an estimated 0.2% were hospitalizations in which the patient suffered from IBD (unweighted *N* = 1015, weighted *N* = 1976). Hospitalizations of patients suffering from IBD were primarily patients suffering from ulcerative colitis (UC; 69.8%, unweighted *N* 739, weighted *N* 1375. 95% CI 1311–1440). Hospitalizations of patients suffering from Crohn’s disease (CD) accounted for 30.2% of hospitalizations for SG of patients suffering from obesity and IBD (unweighted *N* 275, weighted *N* 596, 95% CI 544–647).

### Baseline Demographics

Prior to matching, the median age was higher for hospitalizations of patients suffering from IBD (46 years vs. 43 years, *p* < 0.001). Biological sex was similar across IBD status (81.8% female vs 79.5% female, *p* = 0.086). A BMI range of 40–45 kg/m^2^ was the most frequent BMI category, although it was higher in the presence of IBD (36.9% vs. 32%, *p* = 0.004). Hypertension was the most common comorbidity (57.3% vs. 51.8%, *p* = 0.001) followed by diabetes (28.9% vs. 25.2%, *p* = 0.016), depression (28.4% vs. 19.1%, *p* < 0.001), and chronic pulmonary disease (23.7% vs. 18.2%, *p* < 0.001).

### Propensity Matching

A total of 2030 unweighted SG hospitalizations were successfully matched (1015 IBD; 1015 non-IBD). Following propensity score matching, all covariates had a standardized difference score of less than ± 0.10 (Table 1; Supplemental Figure SI–SII). The median age was 46 (standard difference = 0.01). Females comprised over 80% of hospitalizations (standard difference =  − 0.05). The most frequent BMI range was 40–45 kg/m^2^ (standard difference = 0.06).

**Table 1:** Pre- and post-match descriptives

Descriptives	Pre-match			Post-match		
	IBD: yes	IBD: no	Std. diff	IBD: yes	IBD: no	Std. diff
Age, years	46 [38, 55]	43 [35, 52]	0.22	46 [38, 55]	46 [38, 55]	0.01
Biological sex, %						
Male	18.2	20.5	− 0.08	18.2	19.7	− 0.05
Female	81.8	79.5		81.8	80.3	
BMI, %						
30–34 kg/m^2^	2.1	1.9	0.02	2.1	2.4	− 0.03
35–39 kg/m^2^	21.4	19.0	0.08	21.4	22.6	− 0.04
40–44 kg/m^2^	36.9	32.3	0.14	36.9	34.9	0.06
45–49 kg/m^2^	18.1	20.7	− 0.09	18.1	19.6	− 0.05
50–59 kg/m^2^	15.8	17.6	− 0.07	15.8	14.9	0.04
60+ kg/m^2^	3.4	5.2	− 0.12	3.4	4.4	− 0.07
Discharge quarter IV, %	28.5	28.8	− 0.01	28.5	28.0	0.02
Comorbidities, %						
Depression	28.4	19.1	0.31	28.4	29.2	− 0.02
Diabetes	28.9	25.2	0.12	28.9	28.5	0.01
Hypertension	57.3	51.8	0.16	57.3	57.7	− 0.01
Chronic pulmonary disease	23.7	18.2	0.19	23.7	23.3	0.01
Propensity score	0.0 ± 0.0	0.0 ± 0.0	0.35	0.0 ± 0.0	0.0 ± 0.0	0.00

An * indicates that the result could not be presented per the NRD Data Use Agreement. Data presented as median [interquartile range] or percent. Propensity scores presented as mean ± standard deviation

Standardized differences beyond ± 0.10 were considered to indicate imbalance

### Outcomes

The odds of composite post-operative complication were 48% higher for hospitalizations in which the patient suffered from IBD (11.1 vs. 7.8%; aOR 1.48, 95% CI 1.10 to 2.00, *p* = 0.009; Table 2). For patients suffering from IBD, the odds of post-operative complication were statistically similar between patients suffering from CD and patients suffering from UC (CD 10.0% vs. UC 11.6%, OR 1.19, 95% CI 0.79–1.77, *p* = 0.404). There were no in-hospital deaths noted in either group; moreover, during index SG hospitalization, there were no inpatient deaths for patients carrying an IBD diagnosis and for those not carrying an IBD diagnosis. The most common complication was nausea (4.9% vs. 3.7%, *p* = 0.187; Table SIII). Nausea was the only reportable complication; all other individual complications occurred in 10 or fewer hospitalizations within the IBD or non-IBD cohorts and could not be presented per the NRD Data Use Agreement (Table SIII). No statistically significant difference was observed for all-cause 90-day readmissions (aOR = 1.14; 95% CI 0.72 to 1.81; *p* = 0.570; Table 2). The most common reason for 90-day readmission was esophagitis (16.5%; 95% CI 11.7 to 21.3%). For secondary outcomes, LOS was 2% lower for hospitalizations in which the patient had IBD (95% CI 9% lower to 6% higher; *p* = 0.644; Table 2). Hospital cost was 3% higher for hospitalizations in which the patient had IBD (95% CI 0% higher to 7% higher; *p* = 0.078; Table 2).

**Table 2:** SG post-operative outcomes stratified by the presence of IBD

	IBD: yes	IBD: no	aOR (95% CI)	*p*
Index hospitalization				
In-hospital mortality	*	*	*	*
Complication, %	11.1	7.8	1.48 (1.10–2.00)	0.009
Hospital cost, $	$12,108	$11,732	1.03 (1.00–1.07)	0.078
Length of stay, days	1.5	1.6	0.98 (0.91–1.06)	0.644
90-day readmission, %	6.1	5.4	1.14 (0.72–1.81)	0.570

*aOR* adjusted odds ratio

An * indicated that the results could not be presented per the NRD Data Use Agreement

Data presented as percent or average

## Discussion

In this study, we found that hospitalizations of patients with IBD who underwent SG were associated with significantly higher odds of post-operative in-hospital complications compared to hospitalizations of patients without IBD. Our study is unique in that it uses a large nationwide database specific to the United States to explore the short-term outcomes of SG within a complex set of patients. Patients with IBD pose a unique challenge due to the use of multiple immunosuppressive/immunomodulatory medications. These results have important implications regarding the use of SG in patients with IBD for both healthcare providers and patients. Moreover, our study emphasizes the need for comprehensive preoperative planning, careful patient selection, and meticulous post-operative care for individuals suffering from obesity and IBD.

Surgical procedures may be more challenging for patients with IBD; IBD is associated with chronic inflammation and structural damage and this inflammation and structural damage can lead to anatomical alterations in the gastrointestinal tract thereby increasing the likelihood of post-operative complications [18]. Additionally, the use of immunosuppressive medications might contribute to compromised immune response. Patients with a compromised immune response are predisposed to an increased risk of post-operative complications [19]. The systemic nature of IBD (e.g., dysregulated immune responses) may impair wound healing and increase susceptibility to infection [20]. Several other factors might contribute to increased risk of complications in patients with IBD undergoing SG.

IBD is a rare disease and there are limited large studies available for comparison. The majority of the literature have contrasting results related to complications. Reenaers et al. [21] reported that IBD was not associated with differences in post-operative complications following bariatric surgery. More specifically, Reenaers et al. [21] reported that post-operative complication rates were similar between patients with and without IBD. The primary complications experienced by patients with and without IBD were anemia and ferritin and vitamin B_12_ deficiencies. Similarly, a French database study reported no statistically significant differences in post-operative complications between patients who underwent bariatric surgery with IBD and without IBD [22]. In contrast to the above-mentioned literature, our results showed significantly higher overall complications in patients who underwent SG and also suffered from IBD. The aforementioned discrepancies could be explained by our considerably larger sample size. Additionally, our study was specific to hospitalizations for SG; in contrast, the previous studies also included RNY. Hospitalizations for RNY were excluded from our study because there was an exceedingly low number of obesity procedure hospitalizations of patients with IBD where the obesity procedure was RNY. The inclusion of RNY may increase overall complications as previous studies have shown that RNY is associated with increased complications for patients suffering from UC [23, 24]. Importantly, our results showed that nausea was the most common complication following SG, a clinically minor post-operative complication of SG. Despite higher overall complication rates in patients with IBD, there was no difference in overall LOS or cost. Overall complications are generally implicated in increased LOS and overall cost. The similar LOS between the two cohorts in our study may be explained by the overall benign nature of the complications. It could also be related to the administrative coding used to capture complications within the NRD compared to the coding used in international databases; the NRD is limited by the specificity of ICD-9/10 diagnosis and procedures codes [13].

Our study demonstrated that hospital costs were statistically similar irrespective of whether the patient suffered from IBD. These results contradict findings from a cohort study of 52,782 patients with IBD in the Optum Research Database between 2007 and 2016 which reported $22,987 in annual healthcare-related costs per patient among individuals with IBD compared to $6956 among non-IBD controls [25]. However, it is important to note that the Optum study considered annual healthcare costs whereas this study focused on costs accrued during the time of hospitalization for SG. A retrospective analysis of the NRD identified hospitalizations for IBD and stratified patients by whether they also suffered from obesity; these results demonstrated that patients suffering from IBD and obesity were hospitalized for a greater number of days and incurred higher hospitalization-related costs compared to patients hospitalized for IBD who did not suffer from obesity [26]. Considerations of cost are important as patients suffering from both IBD and obesity may have increased medical costs (e.g., from multiple medications or an increased number of medical appointments). Costs of care should be balanced with long-term outcomes and decreased rate of IBD exacerbations [27, 28]. Any additional financial hardship can subsequently detract from the ability of these patients to afford meaningful dietary and lifestyle changes necessary for durable weight loss after SG.

Patients with IBD considering SG should engage in thorough discussions with the bariatric team to understand the potential risks and benefits. A multidisciplinary approach including gastroenterologists, bariatric surgeons, and other healthcare professionals should be involved to assess the patient’s medical history, disease severity, and surgical candidacy. Preoperative optimization, patient education, personalized treatment plans, and close post-operative follow-up are vital to minimize complications and achieve successful outcomes. The perioperative management of drugs, especially for patients on biologics and steroids, is crucial. Close communication with the gastroenterologist, including the correction of nutritional deficits and improvement of physical condition, can all contribute to successfully navigating the challenges posed by surgery. Prehabilitation in patients with IBD has been based on above mentioned common strategies; specifically, these strategies were recommended in patients undergoing ileocolectomy [29]. Similar guidelines can be employed and followed in patients preparing for bariatric surgery.

### Limitations

Although this study has important implications for the surgical management of obesity in patients with comorbid IBD, the retrospective design and cross-sectional nature of the study restrict the generalizability of our conclusions. Due to the NRD Data Use Agreement and limited sample sizes for individual complications, we were restricted to studying the composite rate of post-operative complications rather than comparing specific adverse outcomes, including IBD-specific complications. Thus, IBD-related complications following SG could not be specifically studied, and therefore the effect that bariatric surgery may have on the disease course of IBD could not be directly stated. Additionally, sample size limitations precluded an investigation of other bariatric surgeries, such as the RNY and duodenal switch. These limitations inhibited the extent to which generalized claims regarding the safety and efficacy of bariatric surgery for patients suffering from IBD could be made. Given the focus on SG, the current study supports specific claims about SG as it relates to its applicability in cases of comorbid IBD. The NRD only provides in-hospital outcomes and the current study focused on in-hospital perioperative complications; neither the prevalence nor the nature of long-term complications could be assessed. Finally, the use of the NRD database also presents certain additional limitations; specifically, the lack of true outpatient SG in the database.

## Conclusion

In conclusion, our results showed that patients suffering from IBD who are hospitalized for SG are at higher risk of overall complications (higher rate). IBD status had no association with post-operative 90-day readmissions or hospital cost and LOS during index hospitalization compared to matched controls without IBD. Therefore, we recommend that patients suffering from IBD may still be considered acceptable candidates for SG provided they receive proper rehabilitation in consideration of their higher overall risk of complications. Future efforts should address comparative long-term post-operative outcomes between various bariatric surgery techniques among patients suffering from IBD.

### Supplementary Information

Below is the link to the electronic supplementary material.

Supplementary file1 (DOCX 171 KB)

## Data Availability

The NRD is publicly available. Information about purchasing the NRD can be found at: https://hcupus.ahrq.gov/tech_assist/centdist.jsp.

## References

[1] Hales CM, Carroll MD, Fryar CD, Ogden CL. Prevalence of obesity and severe obesity among adults: United States, 2017–2018. NCHS data brief [Internet]. 2020;(360):1–8. Available from: https://www.ncbi.nlm.nih.gov/pubmed/32487284. Accessed 8 Feb 2023. 32487284

[2] Artham SM, Lavie CJ, Milani RV, Ventura HO. The obesity paradox: impact of obesity on the prevalence and prognosis of cardiovascular diseases. Postgraduate medicine [Internet]. 2008;120[2]:34–41. Available from: https://www.tandfonline.com/doi/abs/10.3810/pgm.2008.07.1788 . 10.3810/pgm.2008.07.1788 18654066

[3] Ward ZJ, Bleich SN, Long MW, Gortmaker SL. Association of body mass index with health care expenditures in the United States by age and sex. PLoS One [Internet]. 2021;16(3):e0247307. Available from: https://www.ncbi.nlm.nih.gov/pubmed/33760880. Accessed 8 Feb 2023. 10.1371/journal.pone.0247307 PMC7990296 33760880

[4] Xu F, Carlson SA, Liu Y, Greenlund KJ. Prevalence of inflammatory bowel disease among Medicare fee-for-service beneficiaries — United States, 2001−2018. MMWR Morb Mortal Wkly Rept [Internet]. 2021;70(19):698–701. Available from: https://www.ncbi.nlm.nih.gov/pubmed/33983913. Accessed 3 Jul 2023. 10.15585/mmwr.mm7019a2 PMC8118152 33983913

[5] Wang R, Li Z, Liu S, Zhang D. Global, regional and national burden of inflammatory bowel disease in 204 countries and territories from 1990 to 2019: a systematic analysis based on the global burden of disease study 2019. BMJ Open [Internet]. 2023;13(3):e065186. Available from: 10.1136/bmjopen-2022-065186. 10.1136/bmjopen-2022-065186 PMC10069527 36977543

[6] Flores A, Burstein E, Cipher DJ, Feagins LA. Obesity in inflammatory bowel disease: a marker of less severe disease. Dig Dis Sci [Internet]. 2015;60(8):2436–45. Available from: https://link.springer.com/article/10.1007/s10620-015-3629-5 . 10.1007/s10620-015-3629-5 25799938

[7] Bertin B, Desreumaux P, Dubuquoy L. Obesity, visceral fat and Crohnʼs disease. Curr Opin Clin Nutr Metab Care [Internet]. 2010;13[5]:574–80. Available from: https://www.ncbi.nlm.nih.gov/pubmed/20625283. 10.1097/MCO.0b013e32833cf0f4 20625283

[8] Karagiannides I, Pothoulakis C. Substance P, obesity, and gut inflammation. Curr Opin Endocrinol Diabetes Obes [Internet]. 2009;16[1]:47–52. Available from: https://www.ncbi.nlm.nih.gov/pubmed/19104238. 10.1097/MED.0b013e328321306c PMC4404028 19104238

[9] Braga Neto MB, Gregory MH, Ramos GP, Bazerbachi F, Bruining DH, Abu Dayyeh BK, Kushnir VM, Raffals LE, Ciorba MA, Loftus EV, Deepak P. Impact of bariatric surgery on the long-term disease course of inflammatory bowel disease. Inflamm Bowel Dis [Internet]. 2020;26(7):1089–97. Available from: https://www.ncbi.nlm.nih.gov/pubmed/31613968. 10.1093/ibd/izz236 PMC7534455 31613968

[10] Courcoulas AP, Yanovski SZ, Bonds D, Eggerman TL, Horlick M, Staten MA, Arterburn DE. Long-term outcomes of bariatric surgery: a national institutes of health symposium. JAMA Surg [Internet]. 2014;149(12):1323-9. Available from:10.1001/jamasurg.2014.2440. 10.1001/jamasurg.2014.2440 PMC5570469 25271405

[11] Ozsoy Z, Demir E. Which bariatric procedure is the most popular in the world? A bibliometric comparison. Obes Surg [Internet]. 2018;28[8]:2339–52. Available from: https://link.springer.com/article/10.1007/s11695-018-3163-6 . 10.1007/s11695-018-3163-6 29512038

[12] Heshmati K, Lo T, Tavakkoli A, Sheu E. Short-term outcomes of inflammatory bowel disease after Roux-en-Y gastric bypass vs sleeve gastrectomy. J Am Coll Surg [Internet]. 2019;228(6):893,901.e1. Available from: 10.1016/j.jamcollsurg.2019.01.021. 10.1016/j.jamcollsurg.2019.01.021 30797083

[13] NRD Overview. Healthcare Cost and Utilization Project (HCUP). 2022. Agency for Healthcare Research and Quality, Rockville, MD. www.hcup-us.ahrq.gov/nrdoverview.jsp. Accessed January 31, 2023.

[14] ICD-10-CM/PCS MS-DRG v36.0 Definitions Manual [Internet]. Available from: https://www.cms.gov/icd10m/version36-fullcode-cms/fullcode_cms/P0370.html. Accessed 18 Dec 2023.

[15] Elixhauser comorbidity software refined for ICD-10-CM healthcare cost and utilization project (HCUP). 2022. Agency for Healthcare Research and Quality, Rockville, MD. www.hcup-us.ahrq.gov/toolssoftware/comorbidityicd10/comorbidity_icd10.jsp. Accessed February 1, 2023. [Internet] .

[16] United States Bureau of Labor Statistics. CPI inflation calculator. available at: https://www.bls.gov/data/inflation_calculator.htm. Accessed january 31, 2023. [Internet] .

[17] Rao JNK, Scott AJ. On simple adjustments to chi-square tests with sample survey data. Ann Stat [Internet]. 1987;15(1):385–97. Available from: http://www.jstor.org/stable/2241089. Accessed 18 Dec 2023.

[18] Holder-Murray J, Marsicovetere P, Holubar SD. Minimally invasive surgery for inflammatory bowel disease. Inflammatory Bowel Dis [Internet]. 2015;21(6):1443–58. Available from: https://www.ncbi.nlm.nih.gov/pubmed/25989341. Accessed 14 Sept 2023. 10.1097/MIB.0000000000000316 PMC4450898 25989341

[19] Kopylov U, Ben‐Horin S, Zmora O, Eliakim R, Katz LH. Anti‐tumor necrosis factor and postoperative complications in Crohn’s disease: systematic review and meta‐analysis. Inflamm Bowel Diseases [Internet]. 2012;18(12):2404–13. Available from: https://onlinelibrary.wiley.com/doi/abs/10.1002/ibd.22954 . 10.1002/ibd.22954 22467533

[20] Xu X, Liu C, Feng B, Liu Z. Dysregulation of mucosal immune response in pathogenesis of inflammatory bowel disease. World J Gastroenterol: WJG [Internet]. 2014;20(12):3255–64. Available from: http://lib.cqvip.com/qk/84123X/201412/90888889504849524950485049.html. 10.3748/wjg.v20.i12.3255 PMC3964397 24695798

[21] Reenaers C, de Roover A, Kohnen L, Nachury M, Simon M, Pourcher G, Trang-Poisson C, Rajca S, Msika S, Viennot S, Alttwegg R, Serrero M, Seksik P, Peyrin-Biroulet L, Picon L, Bourbao Tournois C, Gontier R, Gilletta C, Stefanescu C, Laharie D, Roblin X, Nahon S, Bouguen G, Carbonnel F, Attar A, Louis E, Coffin B. Bariatric surgery in patients with inflammatory bowel disease: a case-control study from the GETAID. Inflamm Bowel Dise [Internet]. 2022;28[8]:1198–206. Available from: https://search.proquest.com/docview/2581278539. 10.1093/ibd/izab249 34636895

[22] Corbière L, Scanff A, Desfourneaux V, Merdrignac A, Ingels A, Thibault R, Bouguen G, Bergeat D. Outcomes of bariatric surgery in patients with inflammatory bowel disease from a French nationwide database. Br J Surg [Internet]. 2023;110[2]:251–9. Available from: https://www.ncbi.nlm.nih.gov/pubmed/36448229. Accessed 19 Dec 2022. 10.1093/bjs/znac398 36448229

[23] Heshmati K, Lo T, Tavakkoli A, Sheu E. Short-term outcomes of inflammatory bowel disease after Roux-en-Y gastric bypass vs sleeve gastrectomy. J Am Coll Surg [Internet]. 2019;228(6):893,901.e1. Available from: 10.1016/j.jamcollsurg.2019.01.021. 10.1016/j.jamcollsurg.2019.01.021 30797083

[24] Wallhuss A, Ottosson J, Cao Y, Andersson E, Bergemalm D, Eriksson C, Olén O, Szabo E, Stenberg E. Outcomes of bariatric surgery for patients with prevalent inflammatory bowel disease: a nationwide registry-based cohort study. Surgery [Internet]. 2023;174(2):144-51.10.1016/j.surg.2023.04.059. 10.1016/j.surg.2023.04.059 37263879

[25] Park KT, Ehrlich OG, Allen JI, Meadows P, Szigethy EM, Henrichsen K, Kim SC, Lawton RC, Murphy SM, Regueiro M, Rubin DT, Engel-Nitz NM, Heller CA. The cost of inflammatory bowel disease: an initiative from the Crohn’s & Colitis Foundation. Inflamm Bowel Dise [Internet]. 2020;26(1):1–10. Available from: https://www.ncbi.nlm.nih.gov/pubmed/31112238. Accessed 8 Feb 2023. 10.1093/ibd/izz104 PMC7534391 31112238

[26] Nguyen NH, Ohno-Machado L, Sandborn WJ, Singh S. Obesity is independently associated with higher annual burden and costs of hospitalization in patients with inflammatory bowel diseases. Clin Gastroenterol Hepatol [Internet]. 201917(4):709,718.e7. Available from: 10.1016/j.cgh.2018.07.004. 10.1016/j.cgh.2018.07.004 30012429

[27] Coward S, Heitman SJ, Clement F, Hubbard J, Proulx M, Zimmer S, Panaccione R, Seow C, Leung Y, Datta I, Ghosh S, Myers RP, Swain M, Kaplan GG. Ulcerative colitis-associated hospitalization costs: a population-based study. Can J Gastroenterol Hepatol [Internet]. 2015;29(7):357–62. Available from: https://www.ncbi.nlm.nih.gov/pubmed/26079072. Accessed 14 Sept 2023. 10.1155/2015/627370 PMC4610645 26079072

[28] Principi M, Labarile N, Bianchi FP, Contaldo A, Tafuri S, Ierardi E, Di Leo A. The cost of inflammatory bowel disease management matches with clinical course: a single outpatient centre analysis. Int J Environ Res Public Health [Internet]. 2020;17(12):4549. Available from: https://search.proquest.com/docview/2418758708. Accessed 14 Sept 2023. 10.3390/ijerph17124549 PMC7344991 32599816

[29] Sebastian S, Segal JP, Hedin C, Pellino G, Kotze PG, Adamina M, Campmans-Kuijpers M, Davies J, de Vries AC, Casbas AG, El-Hussuna A, Juillerat P, Meade S, Millán M, Spinelli A. ECCO Topical review: roadmap to optimal peri-operative care in IBD. J Crohn's Colitis, [Internet] 2023;17(2):153–169. Available from:10.1093/ecco-jcc/jjac129 10.1093/ecco-jcc/jjac129 36055337

