# Supplementary material for: Bariatric Surgery Outcomes in Patients with Inflammatory Bowel Disease in the United States: An Analysis of the Nationwide Readmissions Database
Source: Obes Surg. 2024 Feb 27;34(4):1279–85. doi: 10.1007/s11695-024-07111-w (PMC11026179; doi:10.1007/s11695-024-07111-w)
Supplement: Supplementary file 1 — Supplementary file1 (DOCX 171 KB) [file 11695_2024_7111_MOESM1_ESM.docx]

| **Table SI.** ICD-9/10 diagnosis and procedure codes for inclusion criteria | | |
| --- | --- | --- |
| Diagnosis | ICD-9-CM | ICD-10-CM |
| **Irritable Bowel Disease** |  |  |
| Crohn’s Disease | 555.0, 555.1, 555.2 555.9 | K50.- |
| Ulcerative Colitis | 556.0-556.6, 556.8, 556.9 | K51.- |
| **History of Bariatric Surgery** | V45.86, V45.75 | Z98.84, Z90.3 |
| **Body Mass Index** |  |  |
| 30 - 34 | V8530-V8534 | Z6830-Z6834 |
| 35 - 39 | V8535-V8539 | Z6835-Z6839 |
| 40 - 44 | V8541 | Z6841 |
| 45 - 49 | V8542 | Z6842 |
| 50 - 59 | V8543 | Z6843 |
| 60+ | V8544, V8545 | Z6844, Z6845 |
| **Complications** |  |  |
| **Bariatric-specific** | 539.- | K95.- |
| **General** |  |  |
| Nausea | 564.3, 578.0, 787.01,  787.02, 787.03, 569.87 | K91.0, K92.0, R11.0, R11.10, R11.11, R11.2, R11.13 |
| Diarrhea | 797.91, 654.4 | R19.7 |
| Dumping Syndrome | 564.2 | K91.1 |
| Malnutrition | 579.3, 579.9, 263.8, 263.9, 263.0, 263.1, 262 | K91.2, K90.9, E46, E44.0, E44.1, E43 |
| Bowel Obstruction | 997.49, 569.71, 569.79,  560.9, 560.1, 537.3,  560.81, 560.89 | K91.3, K91.30, K91.31, K91.32, K91.850, K91.858, K31.5, K56.5, K56.50, K56.51, K56.52, K56.60, K56.600, K566.01, K566.09, K56.69, K56.690, K56.691, K56.699, K56.7, K56.0 |
| Ileus | 560.1, 560.31 | K56.0, K56.7, K56.3 |
| Incisional Hernia | 552.21, 551.1, 553.21 | K43.0, K43.1, K43.2 |
| Blood Loss Anemia | 285.1 | D62 |
| Respiratory | 512.1, 512.2, 518.51,  518.81, 518.84, 799.1, 997.3- | R09.2, J95.81-, J95.82-,  J95.85-, J95.88, J95.89,  J96.0-, J96.2-, J96.9- |
| Post-procedural | 569.9, 997.49, 564.4, 569.89 | K92.9, K91.89, K91.81, K91.82, K91.83, K92.89 |
| Perforation | 998.2, 531.10, 531.20,  532.10, 532.20, 533.10,  533.20, 534.10, 534.20 | K91.71, K91.72, K25.1, K25.2, K26.1, K26.2, K27.1, K27.2, K28.1, K28.2 |
| **Ulceration** |  |  |
| Gastric | 531.00, 531.10, 531.20,  531.30, 531.90 | K25.0, K25.1, K25.2, K25.3, K25.9 |
| Duodenal | 532.00, 532.10, 532.20,  532.30, 532.90 | K26.0, K26.1, K26.2, K26.3, K26.9 |
| Peptic | 533.00, 533.10, 533.20,  533.30, 533.90 | K27.0, K27.1, K27.2, K27.3, K27.9 |
| Gastrojejunal | 534.00, 534.10, 534.20,  534.30, 534.90 | K28.0, K28.1, K28.2, K28.3, K28.9 |
| **Organ Injury** |  |  |
| Spleen | 865.-, 998.11, 998.12, 998.2, 998.13, 997.99 | S36.0-, D78.02, D78.22, D78.32, D78.12, D78.34, D78.8 |
| Liver | 864.- | S36.11- |
| Pancreas | 863.81, 863.82, 863.83, 863.84, 863.91, 863.92, 863.93, 863.94, 998.11, 998.2, 998.12 | S36.2-, E36.02, E36.12, E89.811, E898.21 |
| Stomach | 863.0, 863.1 | S36.3- |
| Small Intestine | 863.2, 863.3 | S36.4- |
| Colon | 863.40, 863.41, 863.42, 863.43, 863.44, 863.46, 863.49, 863.5- | S36.5- |
| Intra-abdominal | 868.-, 869.- | S36.8-, S36.9- |
| Kidney | 866.- | S37.0- |
| Bladder | 867.0, 867.1 | S37.2- |
| **Hemorrhage** |  |  |
| Intraoperative | 998.11, 998.12 | K91.61, K91.62 |
| Post-procedural | 998.11, 998.12 | K91.840, K91.841, K91.870, K91.871 |
| Other | 578.9, 531.00, 531.20, 532.00, 532.20, 533.00, 533.20, 534.00, 534.20, 535.01, 535.31, 535.41, 535.11, 535.21, 535.61, 535.51, 537.83, 537.84 | K92.2, K25.0, K25.2, K26.0, K26.2, K27.0, K27.2, K28.0, K28.2, K29.01, K29.21, K29.31, K29.41, K29.51, K29.61, K29.71, K29.81, K29.91, K31.811, K31.82 |
| **Infection** |  |  |
| Unspecified | 998.51, 998.59 | T81.40XA, T81.49XA, T81.41-, T81.42-, T81.43- |
| Superficial | Not Available | T81.41- |
| Deep Incisional | Not Available | T81.42- |
| Organ Space | Not Available | T81.43- |
| Procedure | ICD-9-PCS | ICD-10-PCS |
| **Sleeve Gastrectomy** | 43.82 | 0DB64Z3 |

**Table SII.** Pre-match unweighted and weighted hospitalization counts

|  | Unweighted | Weighted | | | Percent |  | 95% CI for Weighted Frequency |  |
| --- | --- | --- | --- | --- | --- | --- | --- | --- |
| Index Hospitalization | |  |  | |  |  |  |  |
| IBD: Yes | | 1,015 | 1,974 | | 0.2 | |  | 1,826 – 2,121 |
| IBD: No | | 491,423 | 912,372 | | 99.8 | |  | 886,390 – 938,353 |
| Overall | | 492,438 | 914,345 | | - | |  | 888,312 – 940,379 |

*Note*. Data presented as count or percent.


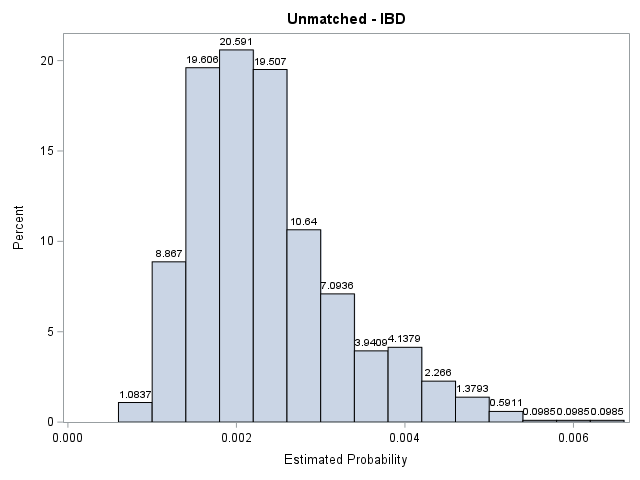

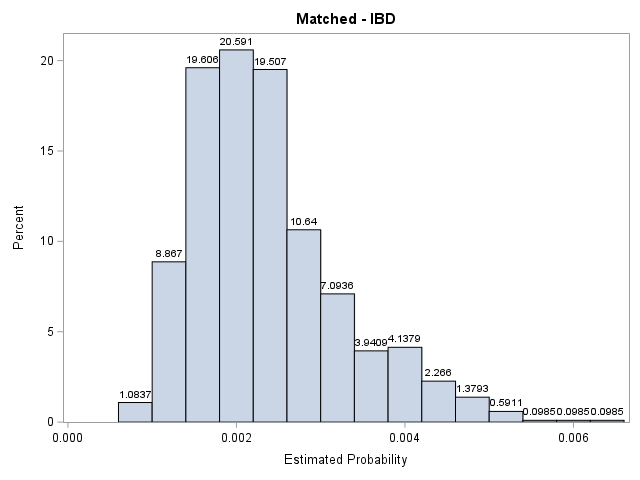

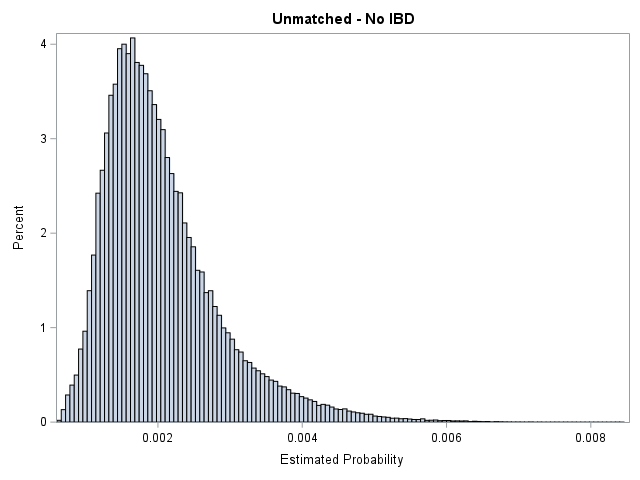

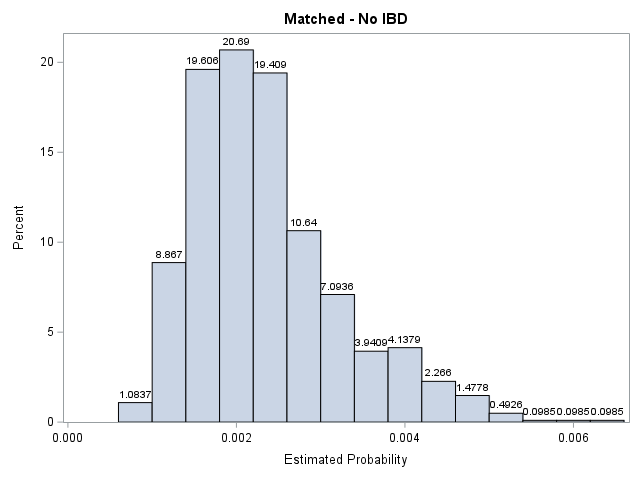


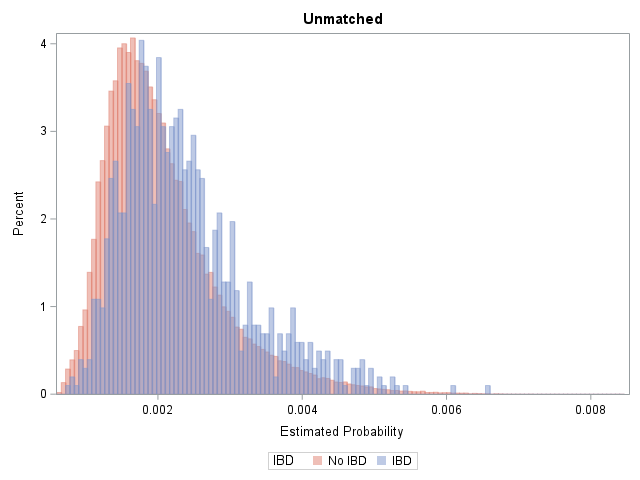

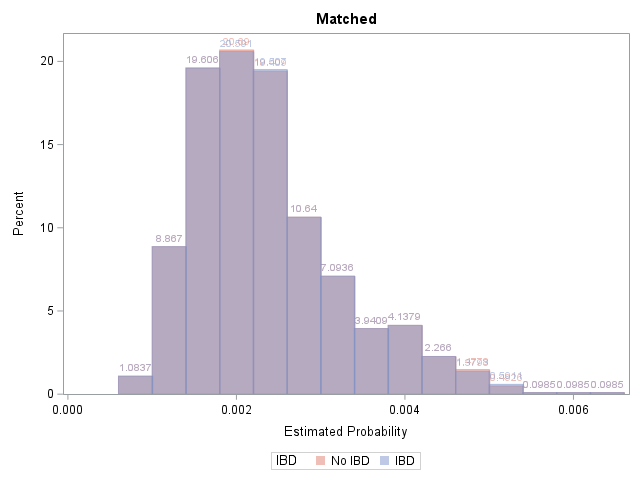


**Supplemental Figure SI.** Distribution of propensity scores for the unmatched (left column) and matched sample (right column) for hospitalizations with (top row) or without IBD (middle row). The bottom row presents an overlay of the top and middle rows.


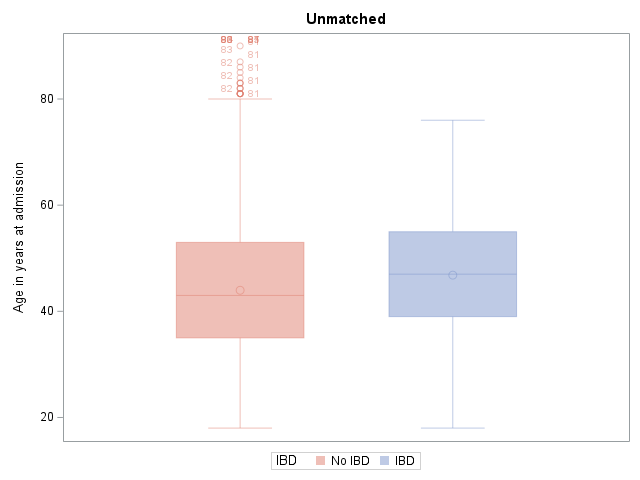

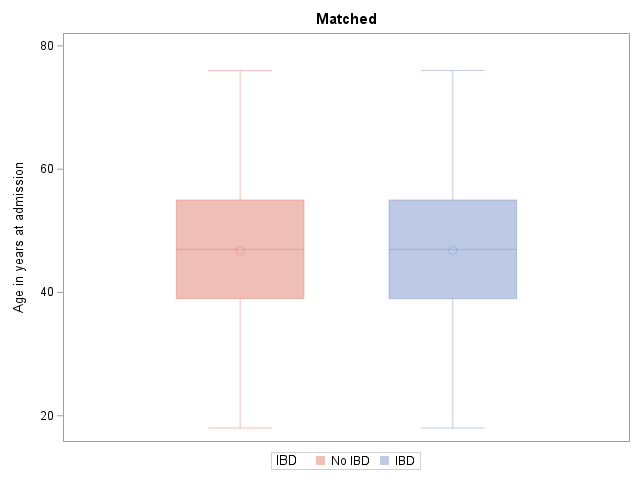


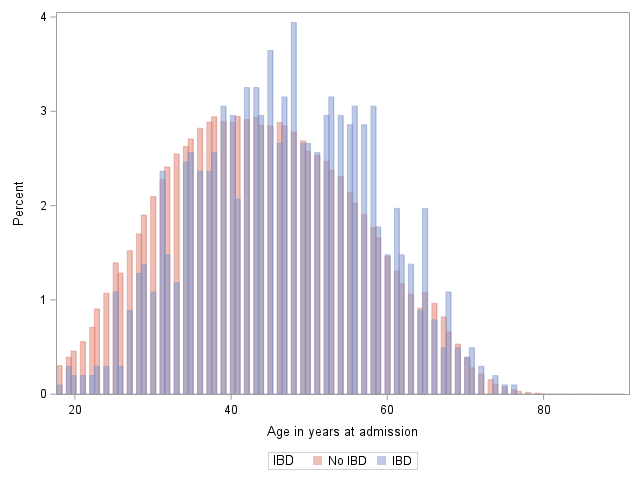

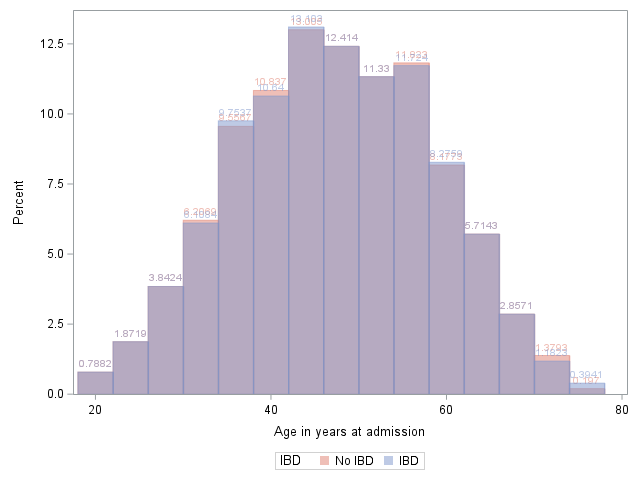


**Supplemental Figure SII.** Boxplot and overlay histograms of age for unmatched sample (left column) and matched sample (right column) for hospitalizations with (blue) or without (red) IBD.

| **Table SIII.** SG post-operative complications stratified by the presence of IBD | | | |
| --- | --- | --- | --- |
| **Complication** | **IBD: Yes** | **IBD: No** | **p** |
| **Any Complication** | 11.1 | 7.8 | 0.009 |
| **Bariatric-specific** | ***** | * | * |
| Bariatric Infection | * | * | * |
| Other Complication | * | * | * |
| **General** | 9.2 | 6.3 | 0.007 |
| Nausea | 4.9 | 3.7 | 0.187 |
| Diarrhea | * | * | * |
| Dumping | * | * | * |
| Malnutrition | * | * | * |
| Bowel Obstruction | * | * | * |
| Ileus | * | * | * |
| Incisional Hernia | 1.2 | * | * |
| Blood Loss Anemia | 1.5 | * | * |
| Respiratory | * | * | * |
| PPC | * | * | * |
| Perforation | * | * | * |
| **Ulceration** | * | * | * |
| Gastric | * | * | * |
| Duodenal | * | * | * |
| Peptic | * | * | * |
| Gastrojejunal | * | * | * |
| **Organ** | * | * | * |
| Spleen | * | * | * |
| Liver | * | * | * |
| Pancreas | * | * | * |
| Stomach | * | * | * |
| Small Intestine | * | * | * |
| Colon | * | * | * |
| Intra-abdominal | * | * | * |
| Kidney | * | * | * |
| Bladder | * | * | * |
| **Hemorrhage** | 1.7 | 1.4 | 0.719 |
| Intraoperative | * | * | * |
| Post Procedural | * | * | * |
| Other | * | * | * |
| Transfusion | 1.0 | * | * |
| **Infection (ICD-10 Only)** | * | * | * |
| Unspecified | * | * | * |
| Superficial | * | * | * |
| Deep Incisional | * | * | * |
| Organ Space | * | * | * |
| *Note*. An * indicates that the result could not be presented per the NRD Data Use Agreement. Data presented as percent. | | | |
